# Camouflaging in a Complex Environment—Octopuses Use Specific Features of Their Surroundings for Background Matching

**DOI:** 10.1371/journal.pone.0037579

**Published:** 2012-05-23

**Authors:** Noam Josef, Piero Amodio, Graziano Fiorito, Nadav Shashar

**Affiliations:** 1 Department of Life Sciences, Ben-Gurion University of the Negev, Eilat Campus, Beer-Sheva, Israel; 2 Stazione Zoologica Anton Dohrn, Napoli, Italy; Institute of Marine Research, Norway

## Abstract

Living under intense predation pressure, octopuses evolved an effective and impressive camouflaging ability that exploits features of their surroundings to enable them to “blend in.” To achieve such background matching, an animal may use general resemblance and reproduce characteristics of its entire surroundings, or it may imitate a specific object in its immediate environment. Using image analysis algorithms, we examined correlations between octopuses and their backgrounds. Field experiments show that when camouflaging, *Octopus cyanea* and *O. vulgaris* base their body patterns on selected features of nearby objects rather than attempting to match a large field of view. Such an approach enables the octopus to camouflage in partly occluded environments and to solve the problem of differences in appearance as a function of the viewing inclination of the observer.

## Introduction

Predation is a strong evolutionary driving force selecting for the development of various defensive mechanisms and behaviors, including cryptic coloration and camouflage [1]. As such, the better the animal matches its background, the less likely it is to be detected by either predator or prey [2]–[8]. However, several empirical studies revealed that cryptic animals do not necessarily match their backgrounds precisely [9], [10]. Cephalopods, particularly octopuses, possess the remarkable ability to change their body patterns to match changes in their visual background (termed crypsis by Endler [11]), categorized and described in detail for *Octopus cyanea* by Hanlon and Messenger [12].Taking a different approach, crypsis has been described as a range of strategies that prevent detection [13]. Background matching is one strategy to achieve crypsis, the principle of which has long been acknowledged (Darwin [14]). In cephalopods, background matching is a dynamic, visually driven process, in which the animal assesses a range of background variables such as contrast, brightness, edge, orientation, and size of objects when deciding what camouflage pattern to display [12], [15]–[29].

To create such a wide variety of body patterns (described in detail by Borreli [30] for various cephalopod species), an octopus uses its sophisticated skin, which contains pigmented chromatophore organs, reflecting iridophores, and light scattering leucophores (reviewed in [12]). These structures confer on octopuses the ability to instantaneously change their body patterns to produce a range of patterns, often described as uniform, mottled, and disruptive, to achieve deceptive and general resemblance ([3], [12], [21], [31], [32]; patterns for different species described by Borreli [30] and references within).

Recent studies [15], [20], [22]–[24], [28], [33]–[35] used artificial substrates, such as checkerboards, to investigate specific visual cues that elicit the various body patterns in cuttlefish. In those studies, the effects of contrast, aspect ratio, shape, and pattern size were investigated, and some of the cues that trigger the camouflage reaction were revealed. These studies also described a response to size-specific cues rather than to aspect ratios or shapes in the visual background, the effects of mean substrate intensity on the disruptive response, and the organism's sensitivity to spatial phase and localized visual edges.

Octopuses possess a single, mid-wavelength visual pigment, making them essentially colorblind ([16], [25], [36], [37] and reviewed in [12]). An octopus's visual cues, which trigger its pattern matching, are fairly different from those perceived by its predators. Yet an octopus needs to present a body-pattern that will conform to its predator's view of the surroundings. Octopuses are preyed upon by a range of animals, including fish and mammals, and they also try to hide from a variety of potential prey [12], each having its own particular visual system. Obviously, in such transformations, mistakes can occur [25]. Furthermore, every camouflaging organism encounters the ‘point of view’ predicament: since the predator often has a different point of view than that of the camouflaging organism, the latter must use the information it gathers from its own position to present a pattern that matches the surroundings as observed by the former. In our case, the perspective of open water predators, such as fish hunting for a hiding octopus, is from above. In contrast, other predators, e.g., moray eels, have fairly low viewing inclinations. Therefore, the octopus may need to present a pattern that differs from the one it obtains from its benthic point of view.

In many cases background matching is thought to refer to matching the average, rather than a single sample, of the background [38], [39]. The immediate surroundings in which octopuses are found are often heterogeneous in vegetation, amount of light, terrain type (whether corals, gravel or sand are visible), color, texture, brightness, contrast, etc. Consequently, the patterns needed for effective concealment are equally diverse, and the task of matching any such object is challenging. The camouflaging animal needs to choose whether to attempt to match a large part of its background or a common, smaller, yet more specific structure in its immediate environment.

In this field study we addressed the following question: does an octopus take into account its entire nearby visual field to achieve what is termed “general resemblance” [3], or does it sample specific features of structures in its surroundings toward what is known as “deceptive resemblance” ([3], reviewed in [31])? Both species examined (*O.cyanea* and *O.vulgaris*) are known as shallow water diurnal predators [40], [41], each with a wide repertoire of body patterns [30]. *O. cyanea* is found throughout the Indo-Pacific region, mostly in coral reef environments, while *O. vulgaris* is common in temperate climate regions where it is frequently found on gravel and in rocky areas.

## Materials and Methods

Given the subjectivity of background matching and its dependence on the viewer, the preferable method for examining camouflage patterns is through an objective and automated image analysis algorithm [42]–[45]. Our experience with such algorithms applied to artificial patterns in controlled environment experiments led us to modify and apply the algorithm used in this study to images of camouflaged, free ranging octopuses and their natural surroundings.

This study was carried out on non-endangered species under the supervision of the Israeli Nature Reserve Authority (Israeli Nature Reserve Authority permit #2010/37233). All necessary permits were obtained for the described field studies under the supervision of the Ben-Gurion University ethics committee under N.J.'s certification of authorization and in accordance with the recommendations in the guide for animal welfare, according to section 1 of the animal welfare law, 1994.

### Analysis by algorithm

Following Zylinski et al. [45], we used an image analysis algorithm [43] in MATLAB™ to test the means, slopes and intercepts of the Rotational-Averaged two dimensional Fast Fourier Transformation (RA-fft or 1D power spectra) of a selected image or part thereof (See [44], [46] for reviews). Application of the algorithm to a range of images produced a similarity map between the camouflaged octopus and the examined surroundings. The outline of the code (available upon request from N.J.) is as follows: a red, green and blue image was obtained and then converted to grayscale using only the green channel. A Gaussian filter (δ = 2) was then used to reduce high-frequency noise followed by a Top-Hat filter (SE = 25) to correct uneven illumination in the scene. Next a square within the octopus's mantle was sampled, 2D-fast Fourier transformation (2D-fft) was applied, the rotational averages (RA-fft) of the octopus were measured, and the lower 2% of frequencies were ignored to avoid spiking. A log-log power spatial frequency' plot was then generated, and the means, slopes and the intercepts of its linear regression were acquired. Finally, non-normality of each sample was verified by a one sample Kolmogorov-Smirnov test. In cases where the means were not significantly different and showed 90% similarity to the mantle, the 90% similarity in the slope differences is assigned to the central pixel. For the (rare) cases where significantly different means were found, no data is presented.

First, an image of an octopus mantle, clearly seen from its top view and containing no less than 150×150 pixels, was sampled and processed. Next, an area of equal size to the mantle sample is moved across the entire image, shifting one pixel at a time, until the entire image is examined. Differences in the parameters (RA-mean, RA-slope and RA-intercept) of octopus vs. background were calculated for each position, and their values were assigned to the central pixel of the frame. This process produced a difference matrix that we then translated into a similarity map superimposed on the original image (except for an edge whose width was one half the size of the shifting area).

### “Per-pixel” method

High resolution images (>1700×1700 pixels) of octopuses were obtained by SCUBA diving on natural reefs (Eilat, northern Gulf of Aqaba, Red Sea; Capri, southern Gulf of Naples, Tyrrhenian Sea) on sunlit days. Free ranging *O. cyanea* (Gray, 1849) and *O. vulgaris* (Cuvier, 1797) were photographed only when they presented a low/flattened body posture without any apparent movement, as observed from a distance of approximately 2 m for at least 1 min. Even though we are well aware of the ‘point of view dilemma’, in this work we considered the pelagic predator's point of view, and therefore, all images of camouflaged octopuses were taken from above and included their immediate surroundings with at least a 1 m radius around the animal for larger *O. cyanea* or a 0.5 m radius for the smaller *O. vulgaris*.

*O. vulgaris* were photographed at distances of over 3 km from each other. *O. cynea*, who are known to be semi-territorial species [40] that typically stay within 80 m of their den and who in Eilat have high spatial fidelity [47], were each photographed over a period of three years in locations that were a minimum of 150 m from each other. This protocol virtually ensures that all our photographs are of different individuals, but because we were neither able to tag nor individually recognize them, there is a very small chance that an octopus was sampled more than once. Eleven images of different octopuses were used for analysis.

**Figure 1:**
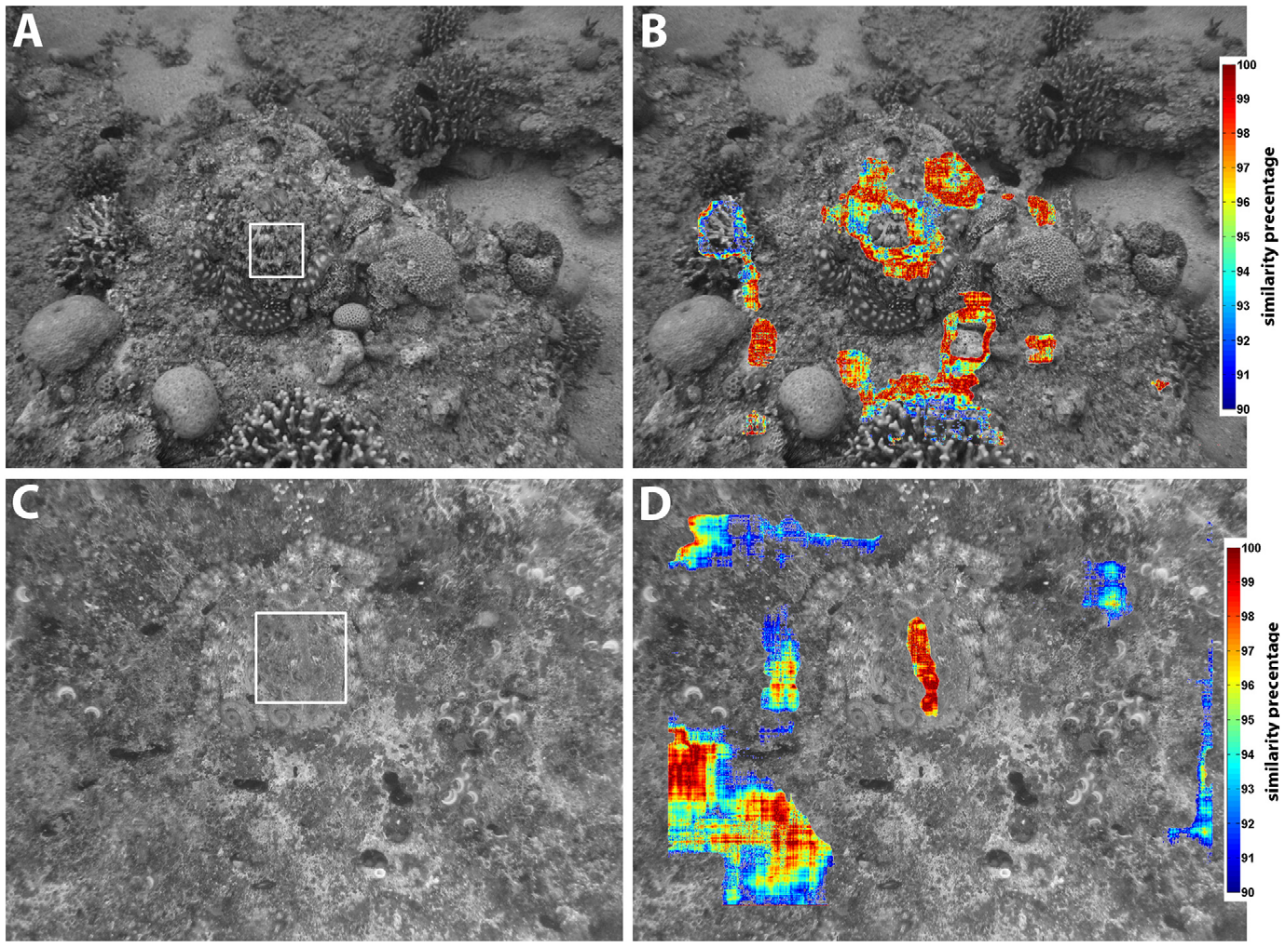
(A,C) Cryptic *O. cyanea* and *O. vulgaris* in their natural habitats. The square defines the mantle sample, which is then compared to the rest of the image. (B, D) Similarity map where areas with resemblance of 90% or higher to the octopus are presented superimposed on the image.

Each image was processed and analyzed with the MATLAB™ code previously described while using the camouflaged octopus's mantle as a reference (Figure 1A, 1C). The mantle sample was then compared to the overall image, and a similarity index map was created as follows:
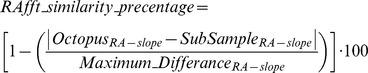
where a low Difference (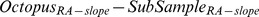) value means high similarity. Only sections of the image with an RA-mean similarity greater than 90% were measured for differences in RA-slopes. Analyzed images are presented as resemblance graphs superimposed over the grayscale image (Figure 1B, 1D).

### “Multi point” method

**Figure 2:**
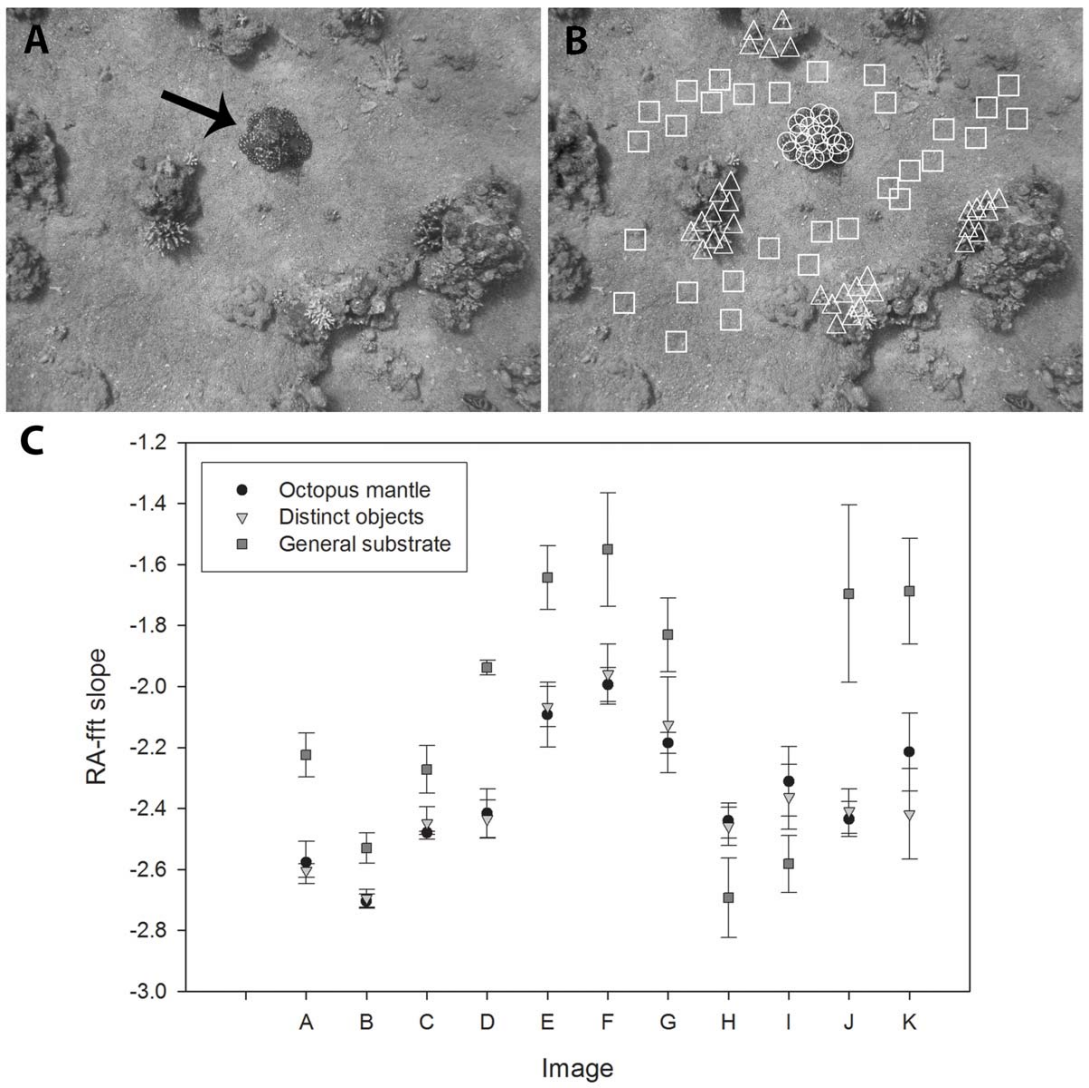
Octopus background matching examined using the multi-point method. (A) Grayscale image of a camouflaging *O. cyanea*, marked with an arrow. (B) Same image as in A showing the 30 samples in each of the three groups marked as: (○) Octopus mantle, (▵) Distinct objects, (□) General substrate. (C) Octopus similarity to background sections using the “Multi-point” method. For each image analyzed (A–K), means± SD are presented for each of the three groups. In all images, the substrate was significantly different from both octopus-mantle and distinctive objects (Kruskal-Wallis test; P<0.01). Different octopuses presented different patterns (Kruskal-Wallis test; P<0.01) and yet resembled specific objects (Mann-Whitney U test; P>0.05). Images A and B are the same images presented in Figures 1 and 2a, respectively.

For further analysis and to obtain statistically analyzable values, we wrote another code to measure and compare the linear regression slopes of log-log power-spatial frequency (following [45]). Taking into account the previous similarity maps (Figure 1B, 1D), we divided our images into three selection types: ‘octopus mantle’, ‘distinct objects’ and ‘general substrate’. We then randomly defined 30 square samples of equal size (100×100 pixels) from each section type (90 points per image) with substantial overlap between them (Figure 2 a–b). Log-log regression slopes of each square were calculated. Slopes from the ‘distinct objects’ and ‘general substrate’ were compared to the ‘octopus mantle’ slopes using a Mann Whitney U test (α = 0.05, n = 30) with statistical software (SPSS™).

## Results

### “Per-pixel” method

Independent images of camouflaged *O. cyanea* in a coral reef environment and *O. vulgaris* on a rocky/algal substrate were analyzed. When squares containing most of the octopuses' mantles were compared to equally sized areas by examining the entire image at single pixel intervals, areas of high resemblance were detected (Figure 1B, 1D). These high resemblance areas matched distinct features and objects in the immediate surroundings but not the entire viewed area or large portions of it. Only when we were absolutely certain that the octopus was situated within a “hidden zone” (i.e, a rock-shelf, large blocking object) from its point of view did we eliminate areas in this zone simply because the octopus could not see them from its position. Even after this elimination, each image examined contained at least one such object or feature that the octopus could indeed see. In most cases (10 out of 11 octopuses, with 1 case not clear), after superimposing the resemblance graph over the original image, we noticed that the areas of high resemblance matched distinct landmarks such as corals, noticeable rocks, patches of unevenly colored sand, or an algae patch whose appearance differed from that of its surroundings.

### “Multi-point”

Analysing the same 11 images of free ranging octopuses using the multi-point method (Figure 2c) showed that a) octopuses displayed body patterns significantly different from each other (Kruskal Wallis test; P<0.01), b) octopus body patterns resembled specific structures in their immediate surroundings (Mann Whitney U test n = 30, P>0.05), and c) such similarity was not found when comparing the octopuses to the ‘general substrate’ sections in the images (P<0.01).

## Discussion

Cephalopod camouflage techniques attract the interest of researchers and the public alike (see [31] for octopus in a reef environment; reviewed in [29]). The patterns they present, generally termed uniform, mottled, or disruptive, are used to achieve deceptive or general resemblance to the octopuses' background or to objects contained within that background [21], [31]. Despite the knowledge acquired about the wide variety of patterns octopuses are capable of producing (reviewed in [30]), there is still the challenge of determining the level of camouflage these patterns provide the animal on a given background [31]. Indeed, the recognition of the “similarity” between two images using a computer program is a known problem in the computer vision field that, due to its complexity, is still not fully understood. This study and previous image analysis research [43], [45], [48], [49] demonstrate that RA-fft slopes are fairly good descriptors for recognizing such similarity. However, it is likely that as research progresses, other and better descriptors will be defined. Knowledge about the visual systems of predators and prey and about image analysis processes in the retina and brain of octopuses will assist in improving our ability to mathematically describe such a similarity.

Previous studies showed that visual information dominates cephalopods' choices of their cryptic body patterns [12], [15]–[18], [20], [22]–[26], [28], [29], [50], [51]. In this study, we examined how much of the available information is being used, i.e., whether an octopus takes into account its entire nearby visual field or samples specific features of selected structures in its surroundings. Our results indicate that both *O. cyanea* and *O. vulgaris* base their body patterns on selected structures rather than on their entire fields of view. It is crucial to emphasize that we do not suggest that octopuses, or any of their predators and prey, do not actually use a mathematical process or function, but rather that RA-fft provides a good estimation as to similarity, and can serve as a proxy to study the features used by the octopuses.

Conventional thinking ( [3], [29], [31] and reference within) is that an octopus attempting to camouflage itself in complex and colorful surroundings typical of coral reefs faces two main options: it can imitate the overall characteristics of its close surroundings or it can choose to imitate a certain object and its characteristics. Hanlon and Messenger [12] alluded to these two options when describing the responses of young cuttlefish to a range of environments, and Hanlon et al. [31] described the moving rock camouflage of an octopus crossing a sandy area. The analysis presented here shows that when camouflaging, an octopus samples specific features of selected structures in its surroundings, i.e., it performs “deceptive resemblance,” sometimes referred to as *element imitation* (as opposed to *object imitation*) [52]. However, the octopus does not imitate the object precisely (in our case, it does not look exactly like any given branching coral), but rather uses key features of the objects common in its surroundings. A possible advantage to such a mechanism is that it can fit a wide range of locations even if the exact level of the match is not perfect. Further research is needed to examine whether using key features of a nearby object can solve the viewing point predicament.
